# Prevalence and associated factors of delayed diagnosis of type 2 diabetes mellitus among adults attending a comprehensive specialized hospital in Central Ethiopia: A facility-based cross-sectional study

**DOI:** 10.1371/journal.pone.0353573

**Published:** 2026-10-07

**Authors:** Million Mohammed Asfaw, Alemu Earsido Addila

**Affiliations:** 1 Department of Surgery, School of Medicine, College of Medicine and Health Sciences, Wachemo University, Hosanna, Ethiopia; 2 Department of Public Health, College of Medicine and Health Sciences, Wachemo University, Hosanna, Ethiopia; Tecnologico de Monterrey, MEXICO

## Abstract

**Background:**

Type 2 diabetes mellitus (T2DM) is a chronic metabolic disorder characterized by insulin resistance and progressive pancreatic β-cell dysfunction, leading to persistent hyperglycemia. Delayed diagnosis is a major public health challenge because many patients are recognized only after developing chronic complications, yet evidence on this problem remains limited in Central Ethiopia.

**Objective:**

To assess the prevalence and associated factors of delayed diagnosis of T2DM among patients attending Wachemo University Nigist Eleni Mohammed Memorial Comprehensive Specialized Hospital (WUNEMMCSH), Central Ethiopia.

**Methods:**

A facility-based cross-sectional study was conducted among 390 adult T2DM patients attending follow-up care at WUNEMMCSH between January 2021 and December 2025 (data collection: February 2026), selected by simple random sampling from medical records. Delayed diagnosis was defined as an interval exceeding three months between the first chart-documented fasting plasma glucose (FPG) ≥126 mg/dL and formal physician-documented diagnosis. Bivariable and multivariable binary logistic regression analyses were performed in SPSS version 25, with significance declared at p < 0.05 using adjusted odds ratios (AOR) and 95% confidence intervals (CI).

**Results:**

The prevalence of delayed diagnosis of T2DM was 25.4% (95% CI: 21.1–29.7). In the multivariable model, rural residence (AOR = 2.37; 95% CI: 1.32–4.24), alcohol consumption (AOR = 3.91; 95% CI: 2.27–6.76, i.e., alcohol users had higher odds of delay than non-users), absence of a history of regular medical check-up (AOR = 3.93; 95% CI: 1.69–9.14), and travel distance to a health facility of one hour or more (AOR = 6.55; 95% CI: 3.16–13.58) were significantly associated with delayed diagnosis. Family history of diabetes, educational status, symptoms at first visit, hypertension, and smoking history were not independently associated with delayed diagnosis in the adjusted model.

**Conclusion:**

Approximately one in four adults with T2DM attending WUNEMMCSH experienced delayed diagnosis. Strengthening opportunistic blood glucose screening, promoting regular preventive check-ups, expanding diagnostic access for rural and geographically distant populations, and targeted outreach for alcohol-using populations are essential to reduce delayed diagnosis and its downstream complications.

## Introduction

Type 2 diabetes mellitus (T2DM) is a chronic metabolic disorder characterized by insulin resistance and progressive β-cell dysfunction, resulting in persistent hyperglycemia. It is among the leading causes of global morbidity and mortality because of its strong association with microvascular complications such as retinopathy, nephropathy, and neuropathy, and macrovascular complications including coronary artery disease, stroke, and peripheral vascular disease [1]. Detecting T2DM while patients remain asymptomatic or only mildly symptomatic creates a critical window for lifestyle modification and pharmacologic therapy that can slow disease progression, reduce complication risk, and lower long-term healthcare costs [2].

Delayed diagnosis of T2DM — a clinically meaningful gap between disease onset and formal recognition — is common across diverse healthcare settings, although its magnitude varies by country, health system, and population subgroup [3]. This delay is driven partly by the prolonged asymptomatic phase of early T2DM and partly by limitations in screening and case-finding practices [4,5]. Even in well-resourced settings a substantial proportion of cases are recognized late: a large electronic health record-based cohort study in the United States found that nearly one-quarter of patients with T2DM remained undiagnosed for a prolonged period before formal confirmation, with limited screening and poor risk prediction identified as key drivers [6]. Prolonged undetected hyperglycemia increases the cumulative burden of micro- and macrovascular complications present at diagnosis and is associated with worse glycemic control, greater comorbidity at presentation, and more intensive, costly management [7–10]. From a systems perspective, missed opportunities for early detection also reduce the effectiveness of broader prevention and chronic disease management programmes [11].

In Ethiopia and other low- and middle-income settings, constraints in screening capacity, diagnostic infrastructure, and community awareness of non-communicable diseases mean that a large proportion of individuals with T2DM are diagnosed only after presenting with advanced complications such as nephropathy, neuropathy, or cardiovascular disease — representing years of undetected hyperglycemia and missed opportunities for early intervention [12–14]. This problem is compounded by the prolonged asymptomatic period typical of early T2DM, during which classic osmotic symptoms such as polyuria and polydipsia are mild or absent, so that patients rarely seek care on the basis of symptoms alone [15]; low health literacy, reliance on traditional medicine, and the absence of routine preventive check-ups further reinforce a reactive, illness-driven pattern of healthcare-seeking [16]. A systematic review and meta-analysis of Ethiopian studies identified obesity, physical inactivity, advanced age, hypertension, and urbanization as major contributors to the diabetes burden, alongside limited screening services and poor public awareness as drivers of delayed case identification [17], and other Ethiopian facility-based studies similarly report a substantial burden of undiagnosed or late-recognized diabetes [18]. Locally, health facilities such as WUNEMMCSH serve large catchment populations with high proportions of rural residents who face geographic, financial, and cultural barriers to timely care, and many individuals live with undetected hyperglycemia for years before irreversible complications develop [19,20].

The rationale for this study arose directly from clinical practice. As a General and Endocrine Surgeon at WUNEMMCSH, the lead author regularly manages patients presenting with advanced diabetic complications — including Charcot foot, diabetic foot with osteomyelitis, and necrotizing fasciitis — many of whom present only after years of undiagnosed or poorly managed T2DM. This recurring surgical burden provided firsthand evidence of a systematic upstream diagnostic gap at the primary care level and motivated an effort to quantify and characterize the diagnostic delay that drives much of the downstream surgical and medical burden encountered at this institution.

Although several studies have assessed diabetes prevalence and associated risk factors in Ethiopia, most have focused on disease prevalence, glycemic control [21], or complications rather than specifically on the timing of diagnosis and its determinants, and evidence from Central and Southern Ethiopia in particular remains scarce [22,23]. Variation in study settings, operational definitions, and methodologies across the existing literature has also limited comparability of findings. Identifying the local prevalence and determinants of delayed diagnosis is essential for designing targeted opportunistic screening protocols and strengthening community-based early detection strategies, similar to co-design approaches used to improve early detection in other disease contexts [24]. This study therefore aimed to assess the prevalence and associated factors of delayed diagnosis of T2DM among adult patients attending WUNEMMCSH, Central Ethiopia.

## Methods

### Study design, setting, and period

A facility-based cross-sectional study was conducted at Wachemo University Nigist Eleni Mohammed Memorial Comprehensive Specialized Hospital (WUNEMMCSH), located in Hossana town, the administrative center of Central Ethiopia Regional State, approximately 232 km southwest of Addis Ababa. Established in 1984, WUNEMMCSH serves a catchment population of more than 3.4 million people from Hadiya Zone and surrounding areas, providing comprehensive preventive, curative, rehabilitative, and referral services across multiple departments, including adult and neonatal intensive care, internal medicine, surgery, orthopedics, pediatrics, obstetrics and gynecology, ophthalmology, psychiatry, and dentistry. The hospital recorded approximately 262,917 outpatient visits and 10,881 inpatient admissions in 2023 and employs 996 staff, of whom 721 are health professionals. The study covered medical records for the period January 2021 to December 2025; data collection took place from February 1–28, 2026.

### Study population and eligibility criteria

The source population comprised all adult patients diagnosed with T2DM and receiving antidiabetic treatment at WUNEMMCSH. The study population included those who attended follow-up services between January 1, 2021 and December 31, 2025 and met the eligibility criteria. Patients were included if they had a confirmed T2DM diagnosis, at least one follow-up visit during the study period, had received antidiabetic medication for three months or more, and had a fasting plasma glucose (FPG) measurement recorded in their chart. Patients with type 1 diabetes or gestational diabetes, those with incomplete records lacking key study variables, and those whose charts could not be retrieved were excluded; these exclusions reflect, respectively, the distinct pathophysiology of these other diabetes subtypes, the need to avoid systematic misclassification arising from missing FPG or diagnosis-date data, and chart-retrieval constraints unrelated to any particular patient subgroup.

### Sample size and sampling technique

Sample size was calculated using a single population proportion formula [25], assuming a delayed-diagnosis proportion of 51.4% from a previous related study, a 5% margin of error, and a 95% confidence level (Z = 1.96), yielding 383 participants; after adding 5% to allow for incomplete records, the final sample size was 402. A sampling frame of 805 eligible patient charts was generated from the chronic disease clinic registry, and 402 charts were selected using OpenEpi-generated random numbers. Of these, 390 records (97.0%) met all eligibility criteria and had complete data for analysis after chart-level verification; the remaining 12 were excluded for incomplete records or unretrievable charts, consistent with the exclusion criteria described above.

### Study variables and operational definitions

The outcome variable was delayed diagnosis of T2DM (yes/no). Independent variables spanned five domains: sociodemographic factors (age, sex, residence, educational status, occupation, income), health system–related factors (availability of screening and diagnostic services, referral pathways, staffing), individual and behavioral factors (diabetes awareness, health-seeking behavior, traditional remedy use), clinical factors (comorbidities, symptom status, family history), and lifestyle and metabolic factors (obesity, diet, smoking, alcohol use).

Obesity was determined from central obesity (waist circumference) as documented in the medical chart. Diabetes awareness was assessed retrospectively from documented evidence in the medical record indicating that the patient recognized at least one diabetes risk factor, symptom, or complication, or had a prior diagnosis of diabetes before the current presentation; absence of such documentation was classified as no documented awareness. Travel time to the nearest health facility was based on the estimated one-way travel time recorded in the patient’s chart at intake as recorded by the attending clinician by considering the patient’s place of residence and available means of transportation.

Delayed diagnosis was defined as an interval exceeding three months between the first chart-documented FPG ≥ 126 mg/dL (recorded on at least two separate occasions) [26] and the formal physician-documented diagnosis of T2DM, the latter evidenced either by an explicit diagnostic entry or by initiation of antidiabetic therapy carrying an explicit T2DM label. Early diagnosis was defined as formal diagnosis within three months of the first elevated FPG. An incomplete record was defined as a chart lacking key variables such as FPG values, diagnosis date, or diabetes type.

The three-month threshold was adopted a priori as a pragmatic operational definition rather than a validated clinical cut-point, since no single interval for “delayed” diagnosis is universally established in the literature; comparable studies of diagnostic delay in diabetes and other chronic conditions have used thresholds ranging from one to twelve months depending on setting and data source [3,15]. Three months was considered a clinically meaningful interval because it exceeds the time typically required for confirmatory retesting and initiation of therapy under standard clinical practice, while remaining short enough to capture delays attributable to missed opportunities rather than routine diagnostic work-up. The authors acknowledge that this cut-off is arbitrary to some degree and that alternative thresholds (e.g., one month or six months) could shift the estimated prevalence of delayed diagnosis and, potentially, the magnitude of some associations; sensitivity analyses using alternative thresholds were not performed in this study and are recommended for future work.

### Data collection and quality assurance

Data were abstracted using a structured checklist adapted from previously published instruments and administered electronically via KoboCollect by four BSc-level nurse data collectors under the supervision of one MPH-qualified researcher. The checklist captured sociodemographic, clinical, laboratory, lifestyle, and diagnostic information. To ensure data quality, the abstraction tool was pretested on 5% of the target sample at a nearby facility, data collectors and supervisors received two days of training, and the principal investigator and supervisor conducted daily monitoring with immediate correction of incomplete or inconsistent entries.

### Statistical analysis

Data were exported from KoboCollect to SPSS version 25 for cleaning and analysis. Descriptive statistics (frequencies, percentages, means, and standard deviations) summarized participant characteristics. Bivariable logistic regression was used to screen candidate variables, and those with p < 0.25 were considered for multivariable modeling; the nine-variable model reported here (family history of diabetes, residence, alcohol consumption, educational status, regular medical check-up, distance from health facility, symptoms at first visit, hypertension, and smoking history) was retained to preserve the original pre-specified model structure. Multicollinearity among these predictors was assessed using variance inflation factors (VIF) prior to model fitting; all VIF values were below 1.4, indicating no meaningful multicollinearity. Missingness on these nine variables was negligible (<1%) among the 390 analyzed records, and complete-case analysis was used. Adjusted odds ratios (AORs) with 95% confidence intervals (CIs) were used to quantify associations, with statistical significance set at p < 0.05. Model fit was assessed using the Hosmer–Lemeshow goodness-of-fit test, which indicated adequate fit (χ²(8) = 11.88, p = 0.157). Several additional health-system variables collected in this study (referral delay, missed diagnostic opportunities, availability of screening services, availability of fasting glucose testing, and availability of a trained healthcare provider) showed statistically significant or near-significant associations with delayed diagnosis at the bivariable stage (p < 0.001 to p = 0.21) but were not included in the final multivariable model, in order to preserve consistency with the original nine-variable model structure; this is discussed further as a limitation. During verification against source records, the smoking-history variable in the analytic file was found to be corrupted at the record level: none of the individual-level smoking values for the 390 analyzed records could be independently confirmed against source documentation, and the file-level values were inconsistent with the source-verified aggregate prevalence (139/390, 35.6%; Table 5). Three approaches to handling this variable in the multivariable model were considered. Recoding individual records against a corrected re-entry of the source paper forms was not feasible, because a validated record-level re-entry of this variable had not been completed at the time of this analysis. Excluding smoking history from the multivariable model entirely was considered but not adopted, in order to preserve the pre-specified nine-variable model structure and because smoking history was not a statistically significant predictor in either the originally submitted model or the corrected model (AOR = 0.82, 95% CI: 0.46–1.46, p = 0.494), so its removal would not materially change the model’s substantive conclusions regarding the four significant predictors (residence, alcohol consumption, regular check-up, and distance to facility). The approach ultimately adopted was retention with disclosure: the multivariable model reported in Table 6 uses the smoking-history values as coded in the original analytic file for all 390 records, while the descriptive prevalence in Table 5 reflects the source-verified aggregate figure; this discrepancy is flagged in the Table 6 footnote and discussed as a limitation. Smoking history was not significantly associated with delayed diagnosis in either version of the model, so this data-quality issue is not expected to affect the study’s substantive conclusions.

### Ethical considerations

Ethical clearance was obtained from the Institutional Review Board of Wachemo University College of Medicine and Health Sciences, and permission was secured from WUNEMMCSH administration prior to data collection. As the study used retrospective record review, individual informed consent was waived. Patient confidentiality was maintained throughout; no personal identifiers were recorded, unique codes were assigned to each chart, and all data were stored securely and used solely for research purposes.

## Results

### Sociodemographic characteristics

A total of 390 medical records were included in the final analysis. Slightly more than half of participants were male (203, 52.1%), and the majority were aged 31–45 years (224, 57.4%), followed by 18–30 years (44, 11.3%), 46–60 years (80, 20.5%), and above 60 years (42, 10.8%). The majority identified as Hadiya by ethnicity (291, 74.6%) and were married (245, 62.8%). Educational attainment was fairly evenly split between secondary education and above (150, 38.5%) and primary education (138, 35.4%), while 102 (26.2%) had no formal education; a separate “college and above” category was not distinguished in the source data. Most participants resided in rural areas (247, 63.3%), while 143 (36.7%) were urban residents. Occupationally, 134 (34.4%) were employed and 132 (33.8%) were farmers. More than half of participants (202, 51.8%) reported a monthly family income below 2,000 Ethiopian Birr (ETB) (Table 1).

**Table 1:** Sociodemographic Characteristics of Study Participants at WUNEMMCSH, Central Ethiopia (n = 390).

Characteristic	Category	Frequency (n)	Percent (%)
**Sex**	Male	203	52.1
	Female	187	47.9
**Age**	18-30 years	44	11.3
	31-45 years	224	57.4
	46-60 years	80	20.5
	Above 60 years	42	10.8
**Ethnicity**	Hadiya	291	74.6
	Kembata	45	11.5
	Gurage	17	4.4
	Other	37	9.5
**Residence**	Urban	143	36.7
	Rural	247	63.3
**Marital status**	Single	92	23.6
	Married	245	62.8
	Separated/divorced	31	7.9
	Widowed	22	5.6
**Educational status**	No formal education	102	26.2
	Primary	138	35.4
	Secondary and above	150	38.5
**Occupation**	Farmer	132	33.8
	Employed	134	34.4
	Student	58	14.9
	Merchant	23	5.9
	Daily laborer	24	6.2
	Other	18	4.6
**Religion**	Protestant	223	57.2
	Orthodox	118	30.3
	Muslim	37	9.5
	Other	12	3.1
**Monthly family income (ETB)**	< 2,000	202	51.8
	2,000–4,999	77	19.7
	5,000–9,999	76	19.5
	≥ 10,000	35	9.0

ETB = Ethiopian Birr. Occupation was not documented for 1 participant; percentages for occupation are calculated among participants with available data (n = 389). Educational status categories reflect the three levels recorded in the source data (no formal education, primary, and secondary and above) and are mutually exclusive.

### Health-seeking behavior

Regarding health-seeking behavior, 231 (59.2%) participants had a documented history of prior blood glucose screening and 108 (27.7%) had used traditional remedies. Only 69 (17.7%) had a history of routine medical check-up, and just 91 (23.3%) demonstrated good awareness of diabetes symptoms. Most participants (276, 70.8%) lived an hour or more from the nearest health facility (Table 2).

**Table 2:** Health-seeking behavior characteristics of study participants at WUNEMMCSH, Central Ethiopia (n = 390).

Characteristic	Category	Frequency (n)	Percent (%)
**Previous blood glucose screening**	Yes	231	59.2
	No	159	40.8
**Use of traditional remedies**	Yes	108	27.7
	No	282	72.3
**History of routine medical check-up**	Yes	69	17.7
	No	321	82.3
**Awareness of diabetes symptoms**	Good	91	23.3
	Poor	299	76.7
Distance from health facility (travel time)*	< 1 hour	114	29.2
	≥ 1 hour	276	70.8

*Distance from health facility was recorded in patient charts as estimated travel time to the nearest facility and was operationalized as travel time (<1 hour vs. ≥ 1 hour) in the multivariable analysis (Table 6), reflecting the functional access barrier experienced by patients in this setting.

### Health system–related characteristics

Health system–related barriers were common: 233 (59.7%) records indicated no nearby diabetes screening service and 257 (65.9%) no nearby fasting glucose testing; referral delay was documented in 287 (73.6%) records, and missed diagnostic opportunities at earlier visits were documented in 381 (97.7%). Most patients (361, 92.6%) nonetheless had access to a trained healthcare provider (Table 3).

**Table 3:** Health system–related characteristics of study participants at WUNEMMCSH, Central Ethiopia (n = 390).

Characteristic	Category	Frequency (n)	Percent (%)
**Availability of diabetes screening service**	Yes	157	40.3
	No	233	59.7
**Availability of fasting blood glucose test**	Yes	133	34.1
	No	257	65.9
**Referral delay**	Yes	287	73.6
	No	103	26.4
**Missed opportunity for diagnosis**	Yes	381	97.7
	No	9	2.3
**Availability of trained healthcare provider**	Yes	361	92.6
	No	29	7.4

### Clinical characteristics

Clinically, 97 (24.9%) participants had a documented family history of diabetes, 262 (67.2%) had hypertension, and 34 (8.7%) had cardiovascular disease; central obesity was documented in 139 (35.6%). Most participants (320, 82.1%) were asymptomatic at their first healthcare visit. Across the full chart review period, fatigue was the most frequently documented symptom (312, 80.0%), followed by polydipsia (208, 53.3%), weight loss (114, 29.2%), and polyuria (89, 22.8%) (Table 4).

**Table 4:** Clinical characteristics of study participants at WUNEMMCSH, Central Ethiopia (n = 390).

Characteristic	Category	Frequency (n)	Percent (%)
**Family history of diabetes mellitus**	Yes	97	24.9
	No	293	75.1
**Hypertension**	Yes	262	67.2
	No	128	32.8
**Cardiovascular disease**	Yes	34	8.7
	No	356	91.3
**Central obesity documented**	Yes	139	35.6
	No	251	64.4
**Symptoms present at first visit**	Yes	70	17.9
	No	320	82.1
**Polyuria**	Yes	89	22.8
	No	301	77.2
**Polydipsia**	Yes	208	53.3
	No	182	46.7
**Weight loss**	Yes	114	29.2
	No	276	70.8
**Fatigue**	Yes	312	80.0
	No	78	20.0

Symptom frequencies (polyuria, polydipsia, weight loss, fatigue) reflect documentation across the full chart review period, not exclusively at the first visit.

### Lifestyle and metabolic characteristics

With respect to lifestyle factors, 113 (29.0%) participants had a documented history of alcohol use and 139 (35.6%) a history of smoking (Table 5).

**Table 5:** Lifestyle and metabolic characteristics of study participants at WUNEMMCSH, Central Ethiopia (n = 390).

Characteristic	Category	Frequency (n)	Percent (%)
**Alcohol intake**	Yes	113	29.0
	No	277	71.0
**Smoking**	Yes	139	35.6
	No	251	64.4

### Prevalence of delayed diagnosis

Overall, 99 of 390 participants (25.4%; 95% CI: 21.1–29.7) experienced delayed diagnosis of T2DM, while the remaining 291 (74.6%) were diagnosed without delay (Fig 1).

**Fig 1:**
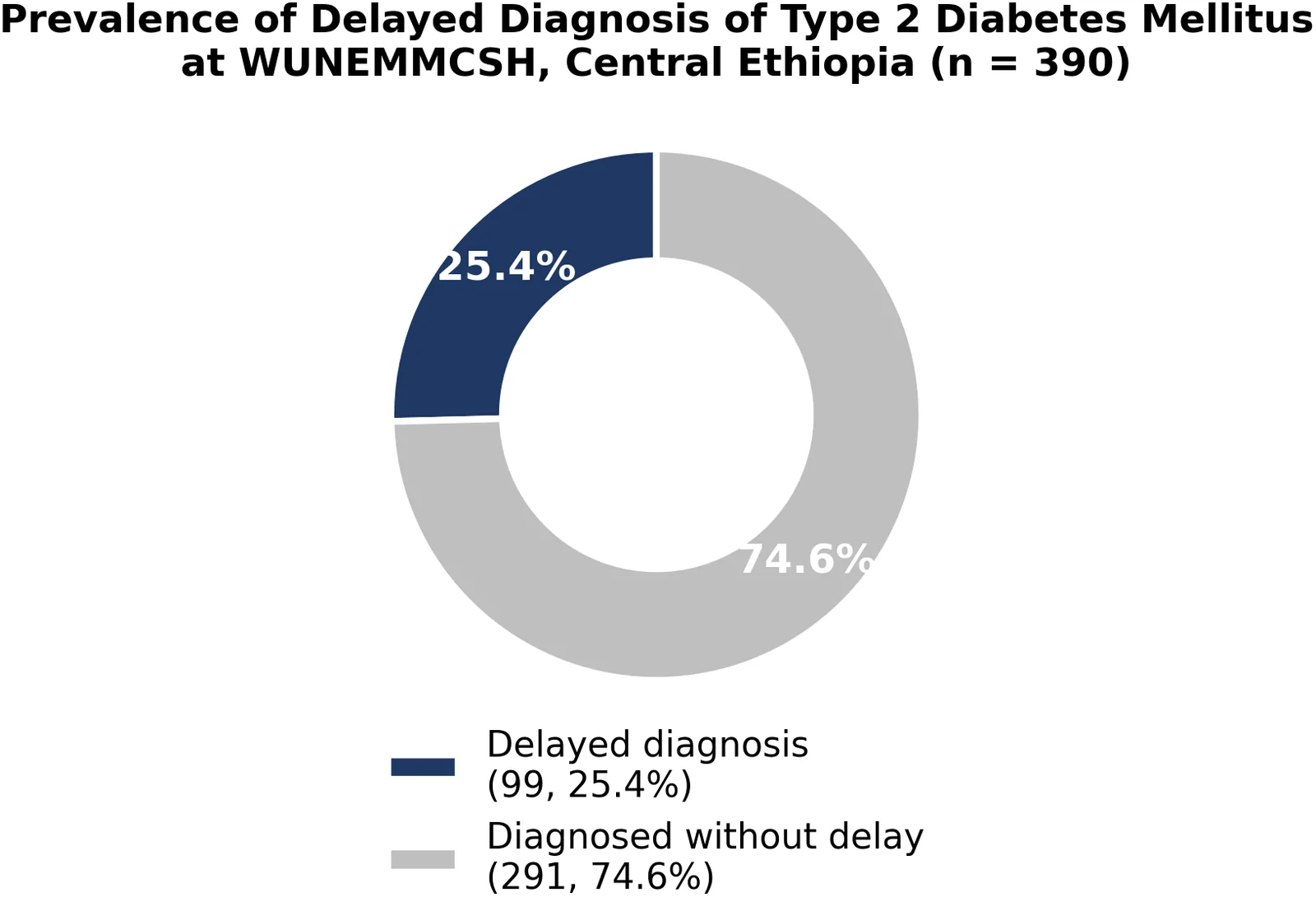
Prevalence of delayed diagnosis of type 2 diabetes mellitus among study participants at WUNEMMCSH, Central Ethiopia (N = 390).

### Factors associated with delayed diagnosis

In multivariable logistic regression, four variables were independently associated with delayed diagnosis (Table 6). Travel distance to a health facility of one hour or more was the strongest predictor (AOR = 6.55; 95% CI: 3.16–13.58; p < 0.001). Alcohol consumption was associated with significantly higher odds of delayed diagnosis relative to non-consumers (AOR = 3.91; 95% CI: 2.27–6.76; p < 0.001, expressed as the odds for alcohol use vs. no use). Absence of a history of regular medical check-up was also associated with higher odds of delay (AOR = 3.93; 95% CI: 1.69–9.14; p = 0.001), as was rural residence (AOR = 2.37; 95% CI: 1.32–4.24; p = 0.004). Family history of diabetes, educational status, symptoms at first visit, hypertension, and smoking history were not independently associated with delayed diagnosis in the adjusted model (p > 0.05 for each).

**Table 6:** Multivariable logistic regression analysis of factors associated with delayed diagnosis of T2DM at WUNEMMCSH, Central Ethiopia (n = 390).

Variable	Category	Delayed Yes n (%)	Delayed No n (%)	COR (95% CI)	AOR (95% CI)	p
**Family history of DM**	Yes (Ref)	21 (21.2)	76 (26.1)	1	1	
	No	78 (78.8)	215 (73.9)	1.31 (0.76–2.27)	1.63 (0.88–3.02)	0.118
**Residence**	Urban (Ref)	22 (22.2)	121 (41.6)	1	1	
	Rural	77 (77.8)	170 (58.4)	2.49 (1.47–4.22)	**2.37 (1.32–4.24)**	**0.004**
**Alcohol consumption**	No (Ref)	49 (49.5)	228 (78.4)	1	1	
	Yes	50 (50.5)	63 (21.6)	3.70 (2.27–5.88)	**3.91 (2.27–6.76)**	**<0.001**
**Educational status**	Secondary and above (Ref)	40 (40.4)	110 (37.8)	1	1	
	No education	31 (31.3)	71 (24.4)	1.41 (0.86–2.33)	1.03 (0.52–2.02)	0.937
	Primary	28 (28.3)	110 (37.8)	0.65 (0.39–1.07)	0.76 (0.42–1.42)	0.396
Regular medical check-up	Yes (Ref)	8 (8.1)	61 (21.0)	1	1	
	No	91 (91.9)	230 (79.0)	3.02 (1.39–6.55)	**3.93 (1.69–9.14)**	**0.001**
Distance from health facility	< 1 hour (Ref)	11 (11.1)	103 (35.4)	1	1	
	≥ 1 hour	88 (88.9)	188 (64.6)	4.38 (2.24–8.58)	**6.55 (3.16–13.58)**	**<0.001**
Symptoms at first visit	Yes (Ref)	21 (21.2)	49 (16.8)	1	1	
	No	78 (78.8)	242 (83.2)	0.75 (0.42–1.33)	0.98 (0.49–1.96)	0.960
Hypertension	No (Ref)	31 (31.3)	97 (33.3)	1	1	
	Yes	68 (68.7)	194 (66.7)	1.10 (0.67–1.79)	1.14 (0.63–2.06)	0.660
Smoking history^†^	Yes (Ref)	74 (74.7)	205 (70.4)	1	1	
	No	25 (25.3)	86 (29.6)	0.81 (0.48–1.35)	0.82 (0.46–1.46)	0.494

*Ref = reference category; COR = crude odds ratio; AOR = adjusted odds ratio; CI = confidence interval. Bold values indicate statistically significant associations (p < 0.05). Values in this table were computed directly from the primary analytic dataset (n = 390). ^†^Smoking-history counts shown in this table reflect the original analytic file; unlike the other variables in this table, they could not be independently re-verified against source records (se*e Statistical Analysis), *and the descriptive prevalence reported in* Table 5 *(139, 35.6%) is the source-verified figure.*

## Discussion

This study found that one in four adults with T2DM attending WUNEMMCSH experienced delayed diagnosis (25.4%; 95% CI: 21.1–29.7), defined as a gap exceeding three months between the first elevated FPG and formal diagnosis. This prevalence is comparable to hospital- and registry-based estimates from the United States and several low- and middle-income countries, where roughly one-quarter of patients with T2DM remained undiagnosed for a prolonged period [6,22], and is consistent with Ethiopian institutional studies reporting delayed diagnosis and late presentation as persistent concerns [17,18,23]. Plausible shared contributing factors include inadequate screening practices, limited community awareness [27], missed opportunities during healthcare visits, and the largely asymptomatic course of early T2DM. The prevalence observed here was lower than reports from some African and Asian settings where delayed diagnosis exceeded one-third of cases [22,28] — a discrepancy that may reflect differences in operational definitions, healthcare accessibility, study design, and public awareness — but higher than estimates below 15% from high-income countries with universal health insurance and routine screening policies [29,30].

Among the four variables independently associated with delayed diagnosis, absence of a history of regular medical check-up carried some of the higher odds (AOR = 3.93; 95% CI: 1.69–9.14), consistent with the predominantly reactive, illness-driven pattern of healthcare utilization reported in other Ethiopian settings [29,31]; regular contact with the health system creates opportunities for incidental glucose testing that symptom-driven care does not. This suggests that individuals without a routine check-up habit represent a high-risk group that may benefit from opportunistic screening integrated into routine outpatient encounters.

Distance from a health facility was the strongest predictor identified (AOR = 6.55; 95% CI: 3.16–13.58), echoing Ethiopian evidence that poor geographic access and transportation costs reduce healthcare utilization [32,33]; in this cohort, 70.8% of participants lived an hour or more from the nearest facility. This finding supports decentralizing glucose screening to health posts and health centers rather than concentrating diagnostic capacity at hospital level.

Alcohol consumption was associated with significantly higher odds of delayed diagnosis relative to non-consumers (AOR = 3.91; 95% CI: 2.27–6.76). This finding warrants caution regarding causal direction, but several plausible, non-mutually-exclusive explanations merit consideration. Alcohol-related comorbidities or acute presentations may draw clinical attention away from metabolic screening, effectively delaying formal T2DM diagnosis even amid frequent healthcare contact [34,35]. Alcohol use may also be associated with reduced engagement in preventive care, minimization of symptoms, or unmeasured confounding by socioeconomic or behavioral factors not captured in this record-based study. Because alcohol exposure and delayed diagnosis were both ascertained from the same cross-sectional chart review, reverse causation cannot be excluded either — for instance, unrelated health difficulties coinciding with alcohol use could independently postpone routine glucose testing. This association should be regarded as hypothesis-generating rather than a basis for causal claims about alcohol’s role in diagnostic delay.

Rural residents had higher odds of delayed diagnosis than urban residents (AOR = 2.37; 95% CI: 1.32–4.24), consistent with the broader literature on lower healthcare access, weaker screening infrastructure, and greater reliance on traditional remedies in rural Ethiopia [28,32]. Notably, rural residents made up the majority of this catchment population (63.3%), underscoring the population-level relevance of this finding. Although rural residence and travel distance may partly overlap, both remained independently significant in the adjusted model, suggesting rural residence captures additional dimensions — health literacy, illness behavior, and livelihood-related economic barriers — beyond physical distance alone. This points to a need for mobile screening units and community health worker-led testing integrated into the Health Extension Programme.

Family history of diabetes, educational status, symptoms at first healthcare visit, hypertension, and smoking history were not independently associated with delayed diagnosis in the adjusted model. The null finding for family history and symptom status is noteworthy given that both are commonly assumed to influence care-seeking or clinical suspicion; it may indicate that, in this setting, structural access barriers (distance, check-up habits) and behavioral factors (alcohol use) outweigh these individual-level clinical cues once accounted for jointly. The null findings for education, hypertension, and smoking similarly contrast with several studies from other sub-Saharan African settings that have reported significant associations for these variables [17,34,36], and may reflect differences in population characteristics, sample size, residual confounding, or limited statistical power for detecting smaller effect sizes within some subgroups; these null results should not be over-interpreted as evidence that these factors are unimportant.

Collectively, these findings indicate that delayed T2DM diagnosis at WUNEMMCSH is shaped primarily by healthcare accessibility (distance, rural residence) and health-seeking behavior (check-up habits, alcohol use), with implications for the risk of chronic complications, recurrent hospitalization, and reduced quality of life among affected patients.

### Strengths and limitations

This study’s principal strengths are its use of secondary clinical record data — minimizing recall bias for several clinical variables — its assessment of multiple domains of determinants (sociodemographic, behavioral, clinical, lifestyle, and health system factors), its multivariable approach to controlling confounding, and an adequately powered sample. Several limitations should also be considered. Reliance on secondary medical record data means that incomplete documentation may have introduced information bias, since the operational definition of delayed diagnosis depended entirely on the completeness of clinical record-keeping. Potential confounders such as dietary practices [37], psychosocial factors, health literacy, provider-related factors, and travel distance recorded in kilometers were not consistently documented and could not be analyzed, leaving residual confounding possible. In addition, because this is a retrospective cross-sectional study in which explanatory variables and the outcome were both ascertained from the same medical record, several explanatory variables — including alcohol consumption, obesity, routine medical check-up behavior, and diabetes awareness — may have been documented at or after the time of diagnosis rather than strictly before it. Temporal sequence between these variables and delayed diagnosis therefore cannot be firmly established, and reverse causation (for example, a diagnosis of T2DM prompting subsequent behavior change or increased health-system contact) remains possible; the associations reported here should accordingly be interpreted as cross-sectional correlations rather than evidence of temporal precedence. Furthermore, several health-system variables (referral delay, missed diagnostic opportunities, availability of screening services, availability of fasting glucose testing, and availability of a trained healthcare provider) showed statistically significant or near-significant bivariable associations with delayed diagnosis (p < 0.001 to p = 0.21) but were not included in the final multivariable model, in order to preserve the original nine-variable model structure; given their strong bivariable associations, their independent contribution should be formally evaluated in future analyses. In addition, the smoking-history variable could not be independently verified at the individual-record level for any of the 390 analyzed records; the multivariable model therefore retains the original file-coded values for this variable rather than the source-verified aggregate figure reported in Table 5, a decision made because record-level recoding was not feasible and because smoking history was non-significant in both versions of the model (see Statistical analysis). Because the study was conducted at a single tertiary institution, findings may not generalize to primary or secondary facilities or to other regions of Ethiopia. Finally, as a facility-based study, it captures only patients who eventually reached WUNEMMCSH; individuals who never sought care — plausibly the most underserved — are systematically excluded, which may lead to underestimation of the true community prevalence of delayed diagnosis.

## Conclusion

Approximately one in four adults with T2DM attending WUNEMMCSH experienced delayed diagnosis, confirming this as a significant public health problem in this setting. Rural residence, alcohol consumption, absence of a history of regular medical check-up, and travel distance of an hour or more to a health facility were independently associated with delayed diagnosis, while family history of diabetes, educational status, symptoms at first presentation, hypertension, and smoking history were not. These findings point to a combination of healthcare accessibility barriers and health-seeking behavior patterns as the principal factors associated with diagnostic delay in this cross-sectional sample. Reducing this burden will require decentralized, community-level screening through health posts, mobile units, and the Health Extension Programme to reach rural and geographically distant populations, together with strategies to promote regular preventive check-ups and targeted outreach for alcohol-using populations. Further prospective, multicenter research is needed to confirm temporal relationships between these factors — including alcohol use — and delayed diagnosis, to evaluate the independent contribution of health-system factors such as referral pathways and screening availability, and to capture variables such as health literacy and dietary practice that were not consistently available in this record-based study.
