# Supplementary material for: Prevalence and associated factors of delayed diagnosis of type 2 diabetes mellitus among adults attending a comprehensive specialized hospital in Central Ethiopia: A facility-based cross-sectional study
Source: PLoS One. 2026 Oct 7;21(10):e0353573. doi: 10.1371/journal.pone.0353573 (PMC13645045; doi:10.1371/journal.pone.0353573)
Supplement: Supplementary file 1 [file pone.0353573.s001.docx]

**Reviewer Comments to the Authors**

**General Comments**

This manuscript examines the prevalence and factors associated with delayed diagnosis of type 2 diabetes mellitus (T2DM) among adults attending a comprehensive specialized hospital in Central Ethiopia. The topic is timely and highly relevant, particularly in low-resource settings where delayed diagnosis contributes substantially to diabetes-related morbidity, mortality, and healthcare costs. The study addresses an important evidence gap in Ethiopia and has potential implications for strengthening diabetes screening and early detection programs.

Overall, the manuscript is well organized, the objectives are clearly stated, and the statistical analyses are generally appropriate. The findings are presented logically and discussed in relation to previous literature. However, several methodological, analytical, and reporting issues require clarification before the manuscript is suitable for publication. In addition, I identified several important numerical inconsistencies that should be carefully verified against the original dataset and statistical output.

**Recommendation: Major Revision**

**Major Comments**

**1. Consistency of the Study Design**

The manuscript submission identifies the study as a "Facility-Based Follow-up Study," whereas the manuscript consistently describes it as a facility-based cross-sectional study. Since the methods clearly indicate a retrospective cross-sectional review of medical records, the study design should be described consistently throughout the title, abstract, and manuscript.

**2. Justification of the Operational Definition**

Delayed diagnosis was defined as an interval greater than three months between the first documented fasting plasma glucose (FPG ≥126 mg/dL) and physician-confirmed diagnosis. Although an operational definition is necessary, the rationale for selecting this cutoff is not adequately justified. The authors should provide an evidence-based justification supported by previous literature or clinical guidelines and discuss whether alternative thresholds could influence the study findings.

**Table 6 counts don't reconcile with the descriptive tables (Tables 1–5) — this is the biggest problem.**

For nearly every variable in the multivariable table, the "Delayed Yes + Delayed No" totals per category don't match the frequencies reported earlier in the manuscript:

**Family history**: Table 6 shows 246 "Yes" / 144 "No" (73+173 and 26+118). Table 4 reports 133 "Yes" / 257 "No". These are essentially reversed and don't match in magnitude either way.

Alcohol intake: Table 6 implies 270 "Yes" / 120 "No" (71+199, 28+92). Table 5 reports 157 "Yes" / 233 "No".

**Regular medical check-up:** Table 6 implies 109 "Yes" / 281 "No" (42+67, 57+224). Table 2 reports 69 "Yes" / 321 "No".

**Symptoms at first visit:** Table 6 implies 154 "Yes" / 236 "No" (75+79, 24+212). Table 4 reports 70 "Yes" / 320 "No".

**Educational status**: Only the "Primary" category (138) matches between Table 1 and Table 6; "Secondary and above" and "College and above" are swapped/mismatched (139 vs 192; 93 vs 37).

These discrepancies suggest either transcription errors, incorrect coding of variables, or presentation of incorrect regression frequencies. The authors should carefully verify Table 6 against the original statistical output and revise the table accordingly.

**4 Temporal Ambiguity**

Because the study is retrospective and cross-sectional, several explanatory variables (e.g., alcohol consumption, obesity, routine medical check-up, and diabetes awareness) may have been measured after diagnosis. Therefore, temporal relationships cannot be established, and reverse causation remains possible. This limitation should be acknowledged more clearly.

**5. Statistical Analysis**

The statistical methods require additional detail. Specifically, the manuscript should report:

- assessment of multicollinearity (e.g., variance inflation factor);
- model diagnostics;
- management of missing data;

Providing these details will improve transparency and reproducibility.

**6. Health System Variables**

Several health-system variables (e.g., referral delay, missed diagnostic opportunities, availability of screening services, and availability of fasting glucose testing) were collected but were not included in the multivariable model. The authors should explain whether these variables were excluded because of statistical non-significance, multicollinearity, missing data, or other methodological considerations.

**7.Direction of the "symptoms" effect is internally contradictory.**

In Table 6, "Yes" (symptoms present) is the reference category (AOR=1) and "No" (asymptomatic) carries AOR=0.23 (95% CI 0.11–0.44) — meaning, as coded, being asymptomatic is associated with lower odds of delay, which is the opposite of the paper's own narrative. The Abstract instead states "AOR=0.23 for symptom presence," and the Discussion says "presence of symptoms was strongly protective" (AOR=0.23) — consistent with each other but inconsistent with how Table 6 is laid out. The authors need to fix either the table's reference category or the surrounding text so the reference group, the AOR, and the narrative all point the same direction. As written, a careful reader can't tell whether symptomatic or asymptomatic patients are actually at higher risk of delay.

**8. Interpretation of Alcohol Consumption**

The finding that non-alcohol consumers had significantly higher odds of delayed diagnosis is unexpected. The explanation proposed in the discussion that alcohol users have more frequent healthcare contact is speculative and not directly supported by the study data. Alternative explanations, including residual confounding, measurement error, and selection bias, should also be considered. The discussion should avoid implying a causal relationship.

**9. Causal Interpretation**

Several statements in the Discussion and Conclusion imply causal relationships (e.g., referring to predictors as "drivers" of delayed diagnosis). Given the observational cross-sectional design, the language should be revised to emphasize associations rather than causation.

**Minor Comments**

**Author name spelling is inconsistent across the document.**

The second author appears as "Alemu Ersido Addila" (abstract), "ALEMU EARSIDO ADILLA" (author order list), and "Alemu Earsido Addila" (byline) — three different spellings of the same name. This should be standardized to match institutional/ORCID records before typesetting

**Abstract**

Include the study period.

**Introduction**

The introduction is informative but could be shortened to improve readability.

Consider incorporating more recent global evidence where appropriate.

**Methods**

Please clarify:

- how obesity was determined;
- how diabetes awareness was measured using medical records;
- how travel time to health facilities was estimated;

**Results**

**Residence**: Consider reporting the distribution of residence in the descriptive tables, as residence appears in the regression model but not in the descriptive results.

**Educational status:** The educational categories are not mutually exclusive. Specifically, "Secondary and above" overlaps with "College and above," making interpretation difficult. The authors should revise the categories to ensure they are mutually exclusive and clearly defined throughout the manuscript.

**Discussion**

The discussion appropriately compares findings with previous literature. However, greater emphasis should be placed on studies from sub-Saharan Africa and on explaining why hypertension, smoking, and educational status were not independently associated with delayed diagnosis.

**Conclusion**

The conclusions are generally supported by the findings but should avoid making strong policy recommendations that imply causal inference.

**Editorial Comments**

- Ensure consistency in terminology throughout the manuscript.
- Standardize abbreviations.
- Carefully proofread the manuscript for grammar and punctuation.
- Verify that all tables, figures, and text report identical numerical values.
- Confirm that tables and figures conform to the journal's formatting requirements.
- Confirm that all references conform to the journal's formatting requirements.
