# Supplementary material for: Prevalence and associated factors of delayed diagnosis of type 2 diabetes mellitus among adults attending a comprehensive specialized hospital in Central Ethiopia: A facility-based cross-sectional study
Source: PLoS One. 2026 Oct 7;21(10):e0353573. doi: 10.1371/journal.pone.0353573 (PMC13645045; doi:10.1371/journal.pone.0353573)
Supplement: Supplementary file 2 [file pone.0353573.s003.docx]

**Response to Reviewers**

**Manuscript title: Prevalence and Associated Factors of Delayed Diagnosis of Type 2 Diabetes Mellitus among Adults Attending a Comprehensive Specialized Hospital in Central Ethiopia: A Facility-Based Cross-Sectional Study**

Dear Editor and Reviewer(s),

We thank the Editor and Reviewer for their careful and constructive evaluation of our manuscript. In response, we undertook a comprehensive revision that included re-verifying every reported figure against the primary analytic dataset and, where discrepancies were identified, against the original source records. Below we respond to each comment point by point. Corresponding changes have been made throughout the revised manuscript, particularly in the Methods, Results (Tables 1–6), Discussion, and Limitations sections.

**MAJOR COMMENTS**

**1. Consistency of the Study Design**

*The manuscript submission identifies the study as a “Facility-Based Follow-up Study,” whereas the manuscript consistently describes it as a facility-based cross-sectional study.*

**Response:** We have corrected this. The study design is now described consistently as a “facility-based cross-sectional study” in the title, abstract, keywords, and throughout the text; the earlier “Follow-up Study” label has been removed.

**2. Justification of the Operational Definition**

*The rationale for the three-month cutoff used to define delayed diagnosis is not adequately justified.*

**Response:** We added a dedicated paragraph in “Study variables and operational definitions” explaining that the three-month threshold was adopted a priori as a pragmatic operational definition, since no single interval is universally validated in the literature; we cite comparable studies using thresholds from one to twelve months, explain why three months was considered clinically meaningful, and explicitly acknowledge that alternative thresholds could shift prevalence and association estimates, recommending sensitivity analysis with alternative cut-points as future work.

**3. Table 6 counts do not reconcile with the descriptive tables (Tables 1–5)**

*For nearly every variable in the multivariable table, the “Delayed Yes + Delayed No” totals per category do not match the frequencies reported in Tables 1–5 (family history, alcohol intake, regular medical check-up, symptoms at first visit, and educational status are all cited as mismatched or reversed).*

**Response:** This was the most serious issue raised, and investigation showed it reflected a genuine data problem rather than a simple table-transcription error: the original multivariable analysis had been run on a corrupted or otherwise incorrect version of the analytic dataset. We re-derived the dataset from the original patient records and re-ran all descriptive and regression analyses. Tables 1–5 have been rebuilt from the verified data, and Table 6 has been completely rebuilt with crude and adjusted odds ratios computed directly on the same verified n=390 sample, so every category total in Table 6 now reconciles exactly with the corresponding descriptive table (e.g., family history: 97 “Yes”/293 “No” in both Table 4 and Table 6; alcohol consumption: 113 “Yes”/277 “No” in both Table 5 and Table 6; regular check-up: 69 “Yes”/321 “No” in both Table 2 and Table 6). The corrected analysis also changed some substantive findings: family history, symptoms at first visit, hypertension, and smoking history are no longer statistically significant in the corrected model, whereas rural residence, alcohol consumption, absence of regular check-up, and travel distance of one hour or more remain independently associated with delayed diagnosis. One exception is disclosed transparently rather than fabricated: the smoking-history variable could not be re-verified at the individual-record level against source documents (see our response to Comment 5 and the Editorial Comments below); Table 6's smoking row now carries an explicit footnote explaining this, and the Statistical Analysis section states the same. Educational status has also been revised to three mutually exclusive categories (no formal education, primary, secondary and above) matching what is actually recorded in the source data — see our response to the Results comment on educational status below.

**4. Temporal Ambiguity**

*Because the study is retrospective and cross-sectional, several explanatory variables may have been measured after diagnosis, so temporal relationships and reverse causation should be acknowledged more clearly.*

**Response:** We expanded the Limitations paragraph to state explicitly that alcohol consumption, obesity, routine check-up behavior, and diabetes awareness were ascertained from the same cross-sectional chart review as the outcome, that temporal sequence between these variables and delayed diagnosis cannot be firmly established, that reverse causation (e.g., a diagnosis prompting subsequent behavior change) remains possible, and that the reported associations should be interpreted as cross-sectional correlations rather than evidence of temporal precedence.

**5. Statistical Analysis**

*The manuscript should report assessment of multicollinearity, model diagnostics, and management of missing data.*

**Response:** All three have been added to the Statistical Analysis section. Multicollinearity was assessed using variance inflation factors for all nine predictors in the multivariable model (all VIF <1.4, indicating no meaningful multicollinearity); model fit was assessed with the Hosmer–Lemeshow goodness-of-fit test (χ²(8)=11.88, p=0.157, indicating adequate fit); and missingness on the nine model variables is reported as negligible (<1% of the 390 analyzed records), with complete-case analysis used.

**6. Health System Variables**

*Several health-system variables were collected but not included in the multivariable model; the authors should explain why.*

**Response:** We added an explanation in the Statistical Analysis section and expanded the Limitations: referral delay, missed diagnostic opportunities, availability of screening services, availability of fasting glucose testing, and availability of a trained healthcare provider showed statistically significant or near-significant bivariable associations with delayed diagnosis (p<0.001 to p=0.21) but were not entered into the final multivariable model, in order to preserve the pre-specified nine-variable model structure used for the primary analysis. We flag their independent contribution as an important direction for future analysis rather than omitting the issue.

**7. Direction of the “symptoms” effect is internally contradictory**

*In Table 6, “Yes” (symptoms present) is the reference category with AOR=0.23 for “No,” implying asymptomatic patients have lower odds of delay — the opposite of the Abstract and Discussion, which state symptom presence was protective (AOR=0.23).*

**Response:** This inconsistency has been resolved by the full data re-verification described in our response to Comment 3. On the corrected dataset, symptoms at first visit is no longer statistically significant (AOR=0.98; 95% CI: 0.49–1.96; p=0.960), so the directional contradiction identified by the reviewer no longer arises. Table 6, the Abstract, and the Discussion are now fully consistent on this variable, and the earlier AOR=0.23 figure no longer appears anywhere in the manuscript.

**8. Interpretation of Alcohol Consumption**

*The finding that non-alcohol consumers had higher odds of delayed diagnosis is unexpected, and the proposed explanation (more frequent healthcare contact among alcohol users) is speculative; alternative explanations should be considered and causal language avoided.*

**Response:** With the corrected dataset, the direction of this association has also changed: alcohol consumption is now a risk factor for delayed diagnosis (AOR=3.91; 95% CI: 2.27–6.76), not protective as in the original submission, which resolves the counter-intuitive direction the reviewer flagged. We also revised the Discussion paragraph on alcohol so that it no longer offers a single speculative mechanism: it now presents several non-mutually-exclusive possibilities (alcohol-related comorbidities or acute presentations diverting clinical attention from metabolic screening, reduced engagement with preventive care, and unmeasured socioeconomic or behavioral confounding), and it explicitly notes that reverse causation cannot be excluded because alcohol exposure and the outcome were both ascertained from the same cross-sectional chart review.

**9. Causal Interpretation**

*Several statements in the Discussion and Conclusion imply causal relationships (e.g., referring to predictors as “drivers”); language should emphasize associations rather than causation given the observational design.*

**Response:** We reviewed the Discussion and Conclusion and revised language that implied causation for our own findings to associational language (“associated with,” “independently associated with”). The word “drivers” now appears only when summarizing a cited prior publication's own conclusions, with the citation attached, and not in reference to our findings.

**MINOR COMMENTS**

**Author name spelling**

*The second author's name appears with three different spellings across the manuscript (“Alemu Ersido Addila,” “ALEMU EARSIDO ADILLA,” “Alemu Earsido Addila”) and should be standardized.*

**Response:** The second author's name has been standardized to “Alemu Ersido Addila” throughout the manuscript, including the byline, matching institutional records. A stray formatting error (a duplicated comma) adjacent to the byline was corrected at the same time.

**Abstract — study period**

*The Abstract should include the study period.*

**Response:** The Methods sentence of the Abstract now explicitly states the study period: “...attending follow-up care at WUNEMMCSH between January 2021 and December 2025 (data collection: February 2026).”

**Introduction**

*The introduction could be shortened, and more recent global evidence could be incorporated.*

**Response:** We reviewed the Introduction and incorporated recent global and regional evidence (including 2025 publications) where relevant. Given the additional methodological detail now required elsewhere in the manuscript (operational-definition justification, expanded Limitations, expanded Statistical Analysis), we have retained the Introduction at close to its original length to preserve context for readers unfamiliar with this setting, but remain glad to trim it further at the Editor's discretion.

**Methods — clarify obesity, diabetes awareness, and travel time**

*Please clarify how obesity was determined, how diabetes awareness was measured using medical records, and how travel time to health facilities was estimated.*

**Response:** All three are now explicitly defined in “Study variables and operational definitions.” Obesity was determined from central obesity (waist circumference) as documented in the medical chart. Diabetes awareness was assessed retrospectively from documented evidence that the patient recognized at least one diabetes risk factor, symptom, or complication, or had a prior diagnosis of diabetes before the current presentation; absence of such documentation was classified as no documented awareness. Travel time to the nearest health facility was based on the estimated one-way travel time recorded in the patient's chart at intake, as assessed by the attending clinician from the patient's place of residence and available means of transportation.

**Results — Residence**

*Consider reporting the distribution of residence in the descriptive tables, since it appears in the regression model but not in the descriptive results.*

**Response:** Residence is now reported in Table 1 (Urban: 143, 36.7%; Rural: 247, 63.3%) in addition to its use in the multivariable model in Table 6, where the category totals now reconcile exactly with Table 1.

**Results — Educational status**

*The educational categories are not mutually exclusive; “Secondary and above” overlaps with “College and above.”*

**Response:** We revised educational status to three mutually exclusive categories that match what is actually recorded in the source data: no formal education, primary, and secondary and above. The overlapping “college and above” category has been removed throughout the manuscript, and a footnote to Table 1 now states explicitly that the categories are mutually exclusive.

**Discussion**

*Greater emphasis should be placed on studies from sub-Saharan Africa and on explaining why hypertension, smoking, and educational status were not independently associated with delayed diagnosis.*

**Response:** We expanded the Discussion paragraph addressing family history, educational status, symptoms, hypertension, and smoking to explicitly compare our null findings with studies from other sub-Saharan African settings, and to offer plausible explanations (differences in population characteristics, sample size and statistical power, residual confounding) while cautioning against over-interpreting these null results as evidence that the factors are unimportant.

**Conclusion**

*Conclusions should avoid strong policy recommendations that imply causal inference.*

**Response:** We revised the Conclusion so that recommendations are explicitly framed as directions supported by the associations observed in this cross-sectional sample, and we added an explicit call for prospective, multicenter research to confirm temporal relationships — including for alcohol use — before stronger causal claims are drawn.

**EDITORIAL COMMENTS**

**Terminology and abbreviations**

*Ensure consistency in terminology throughout the manuscript and standardize abbreviations.*

**Response:** Terminology (e.g., “facility-based cross-sectional study,” “delayed diagnosis”) and abbreviations (T2DM, FPG, AOR, COR, CI, WUNEMMCSH) have been standardized throughout.

**Proofreading**

*Carefully proofread the manuscript for grammar and punctuation.*

**Response:** The manuscript was proofread in full. This included correcting a garbled, duplicated-clause sentence in the operational-definitions paragraph and a formatting error (duplicated comma) in the author byline.

**Numerical consistency across tables, figures, and text**

*Verify that all tables, figures, and text report identical numerical values.*

**Response:** Every numerical value in the abstract, text, and tables was cross-checked against the underlying dataset. This process uncovered two record-level data problems in the analytic file itself (an implausible age value and a corrupted smoking-history variable), which we traced back to the original paper source forms and prior SPSS output and corrected where independent verification was possible. Where verification was not possible — the smoking term within the Table 6 multivariable model only — we chose transparent disclosure via a table footnote and a note in the Statistical Analysis section rather than presenting an unverified number as confirmed; smoking history is not statistically significant in either version of the model, so this does not affect the study's substantive conclusions.

**Table, figure, and reference formatting**

*Confirm that tables and figures conform to the journal's formatting requirements, and that all references conform to the journal's formatting requirements.*

**Response:** Tables have been reformatted for consistent styling (headers, borders, footnotes) throughout the manuscript. We will gladly conform references and tables to any journal-specific style guide the Editor can provide, as the version submitted follows a generic Vancouver-style format pending confirmation of the target journal's specific requirements.

We thank the Editor and Reviewer again for their thorough and constructive feedback, which has substantially improved the accuracy, transparency, and methodological rigor of this manuscript.

Sincerely,

Million Mohammed Asfaw, M.D. (Corresponding author)

Alemu Ersido Addila, Ph.D.
