# Supplementary material for: Prevalence and associated factors of delayed diagnosis of type 2 diabetes mellitus among adults attending a comprehensive specialized hospital in Central Ethiopia: A facility-based cross-sectional study
Source: PLoS One. 2026 Oct 7;21(10):e0353573. doi: 10.1371/journal.pone.0353573 (PMC13645045; doi:10.1371/journal.pone.0353573)
Supplement: Supplementary file 3 [file pone.0353573.s004.docx]

**Response to Editor's Query — Smoking-History Variable**

Manuscript: Prevalence and Associated Factors of Delayed Diagnosis of Type 2 Diabetes Mellitus among Adults Attending a Comprehensive Specialized Hospital in Central Ethiopia: A Facility-Based Cross-Sectional Study

Authors: Million Mohammed Asfaw M.D,MPH , Alemu Earsido Addila Ph.D

**Editor/Reviewer comment:**

*Please clarify precisely how the smoking-history variable was handled in the final multivariable model given that its individual-level values could not be independently verified. Please provide the number of records affected and explain whether exclusion, recoding, or other data-handling procedures were considered.*

**Author response:**

We thank the editor for raising this point and provide the requested clarification below. We have also expanded the manuscript itself (Statistical Analysis and Strengths and Limitations sections, and the Table 6 footnote) so this information is now explicit in the text rather than only in this letter.

**Extent of the issue**: During verification of the analytic file against source records, we found that the smoking-history variable was corrupted at the record level. This is not a partial or isolated data-entry error — none of the individual-level smoking values for any of the 390 analyzed records could be independently confirmed against the original source documentation, and the file-level values were inconsistent with the source-verified aggregate prevalence (139/390, 35.6%, confirmed against an independently generated SPSS output file from the same dataset; see Table 5). All 390 records are therefore affected.

**Options considered:**

(1) Recoding — re-entering the smoking-history values for each record from the original paper-based data collection forms. We considered this the ideal solution, but it was not feasible within the timeframe of this revision because a validated, record-level re-entry of this specific variable had not been completed at the time of analysis.

(2) Exclusion — removing smoking history from the multivariable model entirely. We considered this but did not adopt it, for two reasons: first, doing so would depart from the pre-specified nine-variable model structure that was retained throughout the analysis for consistency with the original submission; second, smoking history was not a statistically significant predictor of delayed diagnosis in either the originally submitted model or the corrected model (AOR = 0.82, 95% CI: 0.46–1.46, p = 0.494), so removing it would not materially change the model's substantive conclusions regarding the four variables that were significant (residence, alcohol consumption, regular medical check-up, and distance to health facility).

(3) Retention with disclosure — the approach we ultimately adopted. The multivariable model reported in Table 6 retains the smoking-history values as originally coded in the analytic file for all 390 records. The descriptive prevalence reported in Table 5, by contrast, reflects the source-verified aggregate figure. We have made this distinction explicit: a footnote under Table 6 flags that the smoking-history counts in that table could not be independently re-verified at the record level, unlike the other variables in the table; the Statistical Analysis section now states the number of records affected (390) and walks through why recoding and exclusion were not adopted; and the Strengths and Limitations section now carries a corresponding limitation statement.

Impact on conclusions: Because smoking history was non-significant in both the original and the source-corrected model, this data-quality limitation does not affect any of the study's substantive conclusions about the factors independently associated with delayed diagnosis. We have been explicit about this throughout the manuscript so that readers can weigh the limitation appropriately.

**Manuscript changes made in response to this comment:**

• Statistical Analysis section: expanded to state the number of affected records (390), and to describe and justify the three data-handling options considered (recoding, exclusion, retention with disclosure).

• Strengths and Limitations section: added a limitation statement summarizing the same information at a level appropriate for that section.

• Table 6 footnote: retained and consistent with the fuller explanation now given in the text.

We appreciate the opportunity to make this handling fully transparent and believe the manuscript is stronger for it.
