# Supplementary material for: Prevalence and associated factors of delayed diagnosis of type 2 diabetes mellitus among adults attending a comprehensive specialized hospital in Central Ethiopia: A facility-based cross-sectional study
Source: PLoS One. 2026 Oct 7;21(10):e0353573. doi: 10.1371/journal.pone.0353573 (PMC13645045; doi:10.1371/journal.pone.0353573)
Supplement: Peer review history

# Peer review: Decision Letter 0

**DOI:** 10.1371/journal.pone.0353573.r001

**Authors:** Mirna Alejandra Gonzalez-Gonzalez

15 Jul 2026

Dear Dr. Asfaw,

Thank you for submitting your manuscript to PLOS ONE. After careful consideration, we feel that it has merit but does not fully meet PLOS ONE’s publication criteria as it currently stands. Therefore, we invite you to submit a revised version of the manuscript that addresses the points raised during the review process.

Please submit your revised manuscript by Sep 13 2026 11:59PM. If you will need more time than this to complete your revisions, please reply to this message or contact the journal office at plosone@plos.org. When you're ready to submit your revision, log on to https://www.editorialmanager.com/pone/ and select the 'Submissions Needing Revision' folder to locate your manuscript file.

- A letter that responds to each point raised by the academic editor and reviewer(s). You should upload this letter as a separate file labeled 'Response to Reviewers'.
- A marked-up copy of your manuscript that highlights changes made to the original version. You should upload this as a separate file labeled 'Revised Manuscript with Track Changes'.
- An unmarked version of your revised paper without tracked changes. You should upload this as a separate file labeled 'Manuscript'.

If you would like to make changes to your financial disclosure, please include your updated statement in your cover letter. Guidelines for resubmitting your figure files are available below the reviewer comments at the end of this letter.

If applicable, we recommend that you deposit your laboratory protocols in protocols.io to enhance the reproducibility of your results. Protocols.io assigns your protocol its own identifier (DOI) so that it can be cited independently in the future. For instructions see: https://journals.plos.org/plosone/s/submission-guidelines#loc-laboratory-protocols. Additionally, PLOS ONE offers an option for publishing peer-reviewed Lab Protocol articles, which describe protocols hosted on protocols.io. Read more information on sharing protocols at https://plos.org/protocols?utm_medium=editorial-email&utm_source=authorletters&utm_campaign=protocols.

As the corresponding author, your ORCID iD is verified in the submission system and will appear in the published article. PLOS supports the use of ORCID, and we encourage all coauthors to register for an ORCID iD and use it as well. Please encourage your coauthors to verify their ORCID iD within the submission system before final acceptance, as unverified ORCID iDs will not appear in the published article. *Only*  the individual author can complete the verification step; PLOS staff *cannot*  verify ORCID iDs on behalf of authors.

We look forward to receiving your revised manuscript.

Kind regards,

Mirna Alejandra Gonzalez-Gonzalez, PhD

Academic Editor

PLOS One

Journal Requirements:

When submitting your revision, we need you to address these additional requirements.

1. Please ensure that your manuscript meets PLOS ONE's style requirements, including those for file naming. The PLOS ONE style templates can be found at

https://journals.plos.org/plosone/s/file?id=wjVg/PLOSOne_formatting_sample_main_body.pdf and

https://journals.plos.org/plosone/s/file?id=ba62/PLOSOne_formatting_sample_title_authors_affiliations.pdf

2. Please provide a complete Data Availability Statement in the submission form, ensuring you include all necessary access information or a reason for why you are unable to make your data freely accessible. If your research concerns only data provided within your submission, please write "All data are in the manuscript and/or supporting information files" as your Data Availability Statement.

3. Please amend either the title on the online submission form (via Edit Submission) or the title in the manuscript so that they are identical.

4. Please amend either the abstract on the online submission form (via Edit Submission) or the abstract in the manuscript so that they are identical.

5. Your ethics statement should only appear in the Methods section of your manuscript. If your ethics statement is written in any section besides the Methods, please move it to the Methods section and delete it from any other section. Please ensure that your ethics statement is included in your manuscript, as the ethics statement entered into the online submission form will not be published alongside your manuscript.

6. If the reviewer comments include a recommendation to cite specific previously published works, please review and evaluate these publications to determine whether they are relevant and should be cited. There is no requirement to cite these works unless the editor has indicated otherwise.

[Note: HTML markup is below. Please do not edit.]

Reviewers' comments:

Reviewer's Responses to Questions

**Comments to the Author**

1. Is the manuscript technically sound, and do the data support the conclusions?

Reviewer #1: Partly

Reviewer #2: No

Reviewer #3: Yes

**********

2. Has the statistical analysis been performed appropriately and rigorously?

Reviewer #1: No

Reviewer #2: No

Reviewer #3: Yes

**********

3. Have the authors made all data underlying the findings in their manuscript fully available?

The PLOS Data policy

Reviewer #1: No

Reviewer #2: No

Reviewer #3: Yes

**********

4. Is the manuscript presented in an intelligible fashion and written in standard English?

Reviewer #1: Yes

Reviewer #2: Yes

Reviewer #3: Yes

**********

Reviewer #1: Thank you so much for the effort on this paper. Please find attached my comments.

• The title doesn't match between the submission system and the manuscript ? Editorial Manager lists it as a "Follow-up Study," but the manuscript header says "Cross-Sectional Study." The Methods clearly describe a cross-sectional retrospective design, so please fix this.

• You state in the submission form that data "ARE FREELY ACCESSIBLE," but the manuscript itself says data are "available from the corresponding author on reasonable request." These two statements contradict each other, and the second one doesn't actually meet PLOS ONE's policy of unrestricted access. Please deposit the dataset in a public repository (Zenodo, Dryad, etc.) with a DOI, and make the form and manuscript consistent.

• You also mention using the Hosmer-Lemeshow test to assess model fit, but I couldn't find the statistic anywhere in Results. Please report it so readers can judge whether the model actually fits.

• I noticed some signs of possible collinearity that aren't addressed. Rural residence and travel distance more than 1 hour overlap conceptually and both stay significant in the adjusted model. Also, "Secondary and above" education jumps from COR=0.17 to AOR=0.85 (95% CI 0.18–3.98) after adjustment. that's a big shift and it's not explained. A VIF check and a short comment would help here.

• Two of your strongest predictors that are "absence of regular check-up" and "absence of prior glucose screening" could just as easily be a consequence of early diagnosis rather than a cause of delayed diagnosis, given the retrospective cross-sectional design. Please discuss this possibility.

• You acknowledge documentation bias for family history, but the same caution isn't applied to symptom status, even though it carries one of the strongest adjusted associations (AOR=0.23). Worth treating both the same way?.

• 97.7% of records show a "missed opportunity for diagnosis," but only 25.4% meet the definition of "delayed diagnosis." That's a large gap and it isn't explained fully, so what separates these two variables operationally?

• The Discussion mostly cites supporting references without comparing effect sizes (AOR/OR) or checking whether "delayed diagnosis" was defined the same way in those studies. Adding that comparison and more details would strengthen the paper's contribution quite a bit.

• A few references are outdated for what they're being used to support. Reference 19 (Nichols et al., 2007) anchors a central comparison with US undiagnosed diabetes prevalence but predates widespread EHR-based screening. References 22 (WHO, 2016) and 30 (Ethiopian FMOH, 2016) are used to support claims about current health-system infrastructure , I would say that definitely more recent sources would serve these points better.

• The sample size was calculated assuming 51.4% prevalence, but you found 25.4%, quite a large difference. Doesn't affect validity, but a brief comment on why the estimates diverged would be useful.

• Minor: please double-check that distance and residence definitions (travel time vs. km) are used consistently throughout.

Reviewer #2: Reviewer Comments to the Authors

General Comments

This manuscript examines the prevalence and factors associated with delayed diagnosis of type 2 diabetes mellitus (T2DM) among adults attending a comprehensive specialized hospital in Central Ethiopia. The topic is timely and highly relevant, particularly in low-resource settings where delayed diagnosis contributes substantially to diabetes-related morbidity, mortality, and healthcare costs. The study addresses an important evidence gap in Ethiopia and has potential implications for strengthening diabetes screening and early detection programs.

Overall, the manuscript is well organized, the objectives are clearly stated, and the statistical analyses are generally appropriate. The findings are presented logically and discussed in relation to previous literature. However, several methodological, analytical, and reporting issues require clarification before the manuscript is suitable for publication. In addition, I identified several important numerical inconsistencies that should be carefully verified against the original dataset and statistical output.

Recommendation: Major Revision

Major Comments

1. Consistency of the Study Design

The manuscript submission identifies the study as a "Facility-Based Follow-up Study," whereas the manuscript consistently describes it as a facility-based cross-sectional study. Since the methods clearly indicate a retrospective cross-sectional review of medical records, the study design should be described consistently throughout the title, abstract, and manuscript.

2. Justification of the Operational Definition

Delayed diagnosis was defined as an interval greater than three months between the first documented fasting plasma glucose (FPG ≥126 mg/dL) and physician-confirmed diagnosis. Although an operational definition is necessary, the rationale for selecting this cutoff is not adequately justified. The authors should provide an evidence-based justification supported by previous literature or clinical guidelines and discuss whether alternative thresholds could influence the study findings.

Table 6 counts don't reconcile with the descriptive tables (Tables 1–5) — this is the biggest problem.

For nearly every variable in the multivariable table, the "Delayed Yes + Delayed No" totals per category don't match the frequencies reported earlier in the manuscript:

Family history: Table 6 shows 246 "Yes" / 144 "No" (73+173 and 26+118). Table 4 reports 133 "Yes" / 257 "No". These are essentially reversed and don't match in magnitude either way.

Alcohol intake: Table 6 implies 270 "Yes" / 120 "No" (71+199, 28+92). Table 5 reports 157 "Yes" / 233 "No".

Regular medical check-up: Table 6 implies 109 "Yes" / 281 "No" (42+67, 57+224). Table 2 reports 69 "Yes" / 321 "No".

Symptoms at first visit: Table 6 implies 154 "Yes" / 236 "No" (75+79, 24+212). Table 4 reports 70 "Yes" / 320 "No".

Educational status: Only the "Primary" category (138) matches between Table 1 and Table 6; "Secondary and above" and "College and above" are swapped/mismatched (139 vs 192; 93 vs 37).

These discrepancies suggest either transcription errors, incorrect coding of variables, or presentation of incorrect regression frequencies. The authors should carefully verify Table 6 against the original statistical output and revise the table accordingly.

4 Temporal Ambiguity

Because the study is retrospective and cross-sectional, several explanatory variables (e.g., alcohol consumption, obesity, routine medical check-up, and diabetes awareness) may have been measured after diagnosis. Therefore, temporal relationships cannot be established, and reverse causation remains possible. This limitation should be acknowledged more clearly.

5. Statistical Analysis

The statistical methods require additional detail. Specifically, the manuscript should report:

• assessment of multicollinearity (e.g., variance inflation factor);

• model diagnostics;

• management of missing data;

Providing these details will improve transparency and reproducibility.

6. Health System Variables

Several health-system variables (e.g., referral delay, missed diagnostic opportunities, availability of screening services, and availability of fasting glucose testing) were collected but were not included in the multivariable model. The authors should explain whether these variables were excluded because of statistical non-significance, multicollinearity, missing data, or other methodological considerations.

7.Direction of the "symptoms" effect is internally contradictory.

In Table 6, "Yes" (symptoms present) is the reference category (AOR=1) and "No" (asymptomatic) carries AOR=0.23 (95% CI 0.11–0.44) — meaning, as coded, being asymptomatic is associated with lower odds of delay, which is the opposite of the paper's own narrative. The Abstract instead states "AOR=0.23 for symptom presence," and the Discussion says "presence of symptoms was strongly protective" (AOR=0.23) — consistent with each other but inconsistent with how Table 6 is laid out. The authors need to fix either the table's reference category or the surrounding text so the reference group, the AOR, and the narrative all point the same direction. As written, a careful reader can't tell whether symptomatic or asymptomatic patients are actually at higher risk of delay.

8. Interpretation of Alcohol Consumption

The finding that non-alcohol consumers had significantly higher odds of delayed diagnosis is unexpected. The explanation proposed in the discussion that alcohol users have more frequent healthcare contact is speculative and not directly supported by the study data. Alternative explanations, including residual confounding, measurement error, and selection bias, should also be considered. The discussion should avoid implying a causal relationship.

9. Causal Interpretation

Several statements in the Discussion and Conclusion imply causal relationships (e.g., referring to predictors as "drivers" of delayed diagnosis). Given the observational cross-sectional design, the language should be revised to emphasize associations rather than causation.

Minor Comments

Author name spelling is inconsistent across the document.

The second author appears as "Alemu Ersido Addila" (abstract), "ALEMU EARSIDO ADILLA" (author order list), and "Alemu Earsido Addila" (byline) — three different spellings of the same name. This should be standardized to match institutional/ORCID records before typesetting

Abstract

Include the study period.

Introduction

The introduction is informative but could be shortened to improve readability.

Consider incorporating more recent global evidence where appropriate.

Methods

Please clarify:

• how obesity was determined;

• how diabetes awareness was measured using medical records;

• how travel time to health facilities was estimated;

Results

Residence: Consider reporting the distribution of residence in the descriptive tables, as residence appears in the regression model but not in the descriptive results.

Educational status: The educational categories are not mutually exclusive. Specifically, "Secondary and above" overlaps with "College and above," making interpretation difficult. The authors should revise the categories to ensure they are mutually exclusive and clearly defined throughout the manuscript.

Discussion

The discussion appropriately compares findings with previous literature. However, greater emphasis should be placed on studies from sub-Saharan Africa and on explaining why hypertension, smoking, and educational status were not independently associated with delayed diagnosis.

Conclusion

The conclusions are generally supported by the findings but should avoid making strong policy recommendations that imply causal inference.

Editorial Comments

• Ensure consistency in terminology throughout the manuscript.

• Standardize abbreviations.

• Carefully proofread the manuscript for grammar and punctuation.

• Verify that all tables, figures, and text report identical numerical values.

• Confirm that tables and figures conform to the journal's formatting requirements.

• Confirm that all references conform to the journal's formatting requirements.

Reviewer #3: Data collection/study covered was done up until 2025, but reported a standpoint of 2023, what happened between 2023 and 2025? Does the 2-year data reflect in the methodology/results?

It was mentioned that 996 staff's were recruited and 721 were health professionals, so, what category of workers do the other's fall into? Kindly state this and how relevant they are to this study or not? What impact do they have on T2DM screening?

What is the impact does the lifestyle/monthly earnings have on the participants/patients with respect to T2DM detection?

Kindly remove hyphens in between sentences, and complete the sentences (re-write) where necessary

**********

what does this mean?). If published, this will include your full peer review and any attached files.

If you choose “no”, your identity will remain anonymous but your review may still be made public.

**Do you want your identity to be public for this peer review?**  For information about this choice, including consent withdrawal, please see our Privacy Policy

Reviewer #1: No

Reviewer #2: No

Reviewer #3: No

**********

[NOTE: If reviewer comments were submitted as an attachment file, they will be attached to this email and accessible via the submission site. Please log into your account, locate the manuscript record, and check for the action link "View Attachments". If this link does not appear, there are no attachment files.]

To ensure your figures meet our technical requirements, please review our figure guidelines: https://journals.plos.org/plosone/s/figures

You may also use PLOS’s free figure tool, NAAS, to help you prepare publication quality figures: https://journals.plos.org/plosone/s/figures#loc-tools-for-figure-preparation.

NAAS will assess whether your figures meet our technical requirements by comparing each figure against our figure specifications.

Attachment Submitted filename: Reviewer Comments to the Authors.docx

# Author comment: Author response to Decision Letter 1

**DOI:** 10.1371/journal.pone.0353573.r002

23 Jul 2026

MAJOR COMMENTS

1. Consistency of the Study Design

The manuscript submission identifies the study as a “Facility-Based Follow-up Study,” whereas the manuscript consistently describes it as a facility-based cross-sectional study.

Response: We have corrected this. The study design is now described consistently as a “facility-based cross-sectional study” in the title, abstract, keywords, and throughout the text; the earlier “Follow-up Study” label has been removed.

2. Justification of the Operational Definition

The rationale for the three-month cutoff used to define delayed diagnosis is not adequately justified.

Response: We added a dedicated paragraph in “Study variables and operational definitions” explaining that the three-month threshold was adopted a priori as a pragmatic operational definition, since no single interval is universally validated in the literature; we cite comparable studies using thresholds from one to twelve months, explain why three months was considered clinically meaningful, and explicitly acknowledge that alternative thresholds could shift prevalence and association estimates, recommending sensitivity analysis with alternative cut-points as future work.

3. Table 6 counts do not reconcile with the descriptive tables (Tables 1–5)

For nearly every variable in the multivariable table, the “Delayed Yes + Delayed No” totals per category do not match the frequencies reported in Tables 1–5 (family history, alcohol intake, regular medical check-up, symptoms at first visit, and educational status are all cited as mismatched or reversed).

Response: This was the most serious issue raised, and investigation showed it reflected a genuine data problem rather than a simple table-transcription error: the original multivariable analysis had been run on a corrupted or otherwise incorrect version of the analytic dataset. We re-derived the dataset from the original patient records and re-ran all descriptive and regression analyses. Tables 1–5 have been rebuilt from the verified data, and Table 6 has been completely rebuilt with crude and adjusted odds ratios computed directly on the same verified n=390 sample, so every category total in Table 6 now reconciles exactly with the corresponding descriptive table (e.g., family history: 97 “Yes”/293 “No” in both Table 4 and Table 6; alcohol consumption: 113 “Yes”/277 “No” in both Table 5 and Table 6; regular check-up: 69 “Yes”/321 “No” in both Table 2 and Table 6). The corrected analysis also changed some substantive findings: family history, symptoms at first visit, hypertension, and smoking history are no longer statistically significant in the corrected model, whereas rural residence, alcohol consumption, absence of regular check-up, and travel distance of one hour or more remain independently associated with delayed diagnosis. One exception is disclosed transparently rather than fabricated: the smoking-history variable could not be re-verified at the individual-record level against source documents (see our response to Comment 5 and the Editorial Comments below); Table 6's smoking row now carries an explicit footnote explaining this, and the Statistical Analysis section states the same. Educational status has also been revised to three mutually exclusive categories (no formal education, primary, secondary and above) matching what is actually recorded in the source data — see our response to the Results comment on educational status below.

4. Temporal Ambiguity

Because the study is retrospective and cross-sectional, several explanatory variables may have been measured after diagnosis, so temporal relationships and reverse causation should be acknowledged more clearly.

Response: We expanded the Limitations paragraph to state explicitly that alcohol consumption, obesity, routine check-up behavior, and diabetes awareness were ascertained from the same cross-sectional chart review as the outcome, that temporal sequence between these variables and delayed diagnosis cannot be firmly established, that reverse causation (e.g., a diagnosis prompting subsequent behavior change) remains possible, and that the reported associations should be interpreted as cross-sectional correlations rather than evidence of temporal precedence.

5. Statistical Analysis

The manuscript should report assessment of multicollinearity, model diagnostics, and management of missing data.

Response: All three have been added to the Statistical Analysis section. Multicollinearity was assessed using variance inflation factors for all nine predictors in the multivariable model (all VIF <1.4, indicating no meaningful multicollinearity); model fit was assessed with the Hosmer–Lemeshow goodness-of-fit test (χ²(8)=11.88, p=0.157, indicating adequate fit); and missingness on the nine model variables is reported as negligible (<1% of the 390 analyzed records), with complete-case analysis used.

6. Health System Variables

Several health-system variables were collected but not included in the multivariable model; the authors should explain why.

Response: We added an explanation in the Statistical Analysis section and expanded the Limitations: referral delay, missed diagnostic opportunities, availability of screening services, availability of fasting glucose testing, and availability of a trained healthcare provider showed statistically significant or near-significant bivariable associations with delayed diagnosis (p<0.001 to p=0.21) but were not entered into the final multivariable model, in order to preserve the pre-specified nine-variable model structure used for the primary analysis. We flag their independent contribution as an important direction for future analysis rather than omitting the issue.

7. Direction of the “symptoms” effect is internally contradictory

In Table 6, “Yes” (symptoms present) is the reference category with AOR=0.23 for “No,” implying asymptomatic patients have lower odds of delay — the opposite of the Abstract and Discussion, which state symptom presence was protective (AOR=0.23).

Response: This inconsistency has been resolved by the full data re-verification described in our response to Comment 3. On the corrected dataset, symptoms at first visit is no longer statistically significant (AOR=0.98; 95% CI: 0.49–1.96; p=0.960), so the directional contradiction identified by the reviewer no longer arises. Table 6, the Abstract, and the Discussion are now fully consistent on this variable, and the earlier AOR=0.23 figure no longer appears anywhere in the manuscript.

8. Interpretation of Alcohol Consumption

The finding that non-alcohol consumers had higher odds of delayed diagnosis is unexpected, and the proposed explanation (more frequent healthcare contact among alcohol users) is speculative; alternative explanations should be considered and causal language avoided.

Response: With the corrected dataset, the direction of this association has also changed: alcohol consumption is now a risk factor for delayed diagnosis (AOR=3.91; 95% CI: 2.27–6.76), not protective as in the original submission, which resolves the counter-intuitive direction the reviewer flagged. We also revised the Discussion paragraph on alcohol so that it no longer offers a single speculative mechanism: it now presents several non-mutually-exclusive possibilities (alcohol-related comorbidities or acute presentations diverting clinical attention from metabolic screening, reduced engagement with preventive care, and unmeasured socioeconomic or behavioral confounding), and it explicitly notes that reverse causation cannot be excluded because alcohol exposure and the outcome were both ascertained from the same cross-sectional chart review.

9. Causal Interpretation

Several statements in the Discussion and Conclusion imply causal relationships (e.g., referring to predictors as “drivers”); language should emphasize associations rather than causation given the observational design.

Response: We reviewed the Discussion and Conclusion and revised language that implied causation for our own findings to associational language (“associated with,” “independently associated with”). The word “drivers” now appears only when summarizing a cited prior publication's own conclusions, with the citation attached, and not in reference to our findings.

MINOR COMMENTS

Author name spelling

The second author's name appears with three different spellings across the manuscript is “ALEMU EARSIDO ADILLA,” “Alemu Earsido Addila”) and should be standardized.

Response: The second author's name has been standardized to “Alemu Earsido Addila” throughout the manuscript, including the byline, matching institutional records. A stray formatting error (a duplicated comma) adjacent to the byline was corrected at the same time.

Abstract — study period

The Abstract should include the study period.

Response: The Methods sentence of the Abstract now explicitly states the study period: “...attending follow-up care at WUNEMMCSH between January 2021 and December 2025 (data collection: February 2026).”

Introduction

The introduction could be shortened, and more recent global evidence could be incorporated.

Response: We reviewed the Introduction and incorporated recent global and regional evidence (including 2025 publications) where relevant. Given the additional methodological detail now required elsewhere in the manuscript (operational-definition justification, expanded Limitations, expanded Statistical Analysis), we have retained the Introduction at close to its original length to preserve context for readers unfamiliar with this setting, but remain glad to trim it further at the Editor's discretion.

Methods — clarify obesity, diabetes awareness, and travel time

Please clarify how obesity was determined, how diabetes awareness was measured using medical records, and how travel time to health facilities was estimated.

Response: All three are now explicitly defined in “Study variables and operational definitions.” Obesity was determined from central obesity (waist circumference) as documented in the medical chart. Diabetes awareness was assessed retrospectively from documented evidence that the patient recognized at least one diabetes risk factor, symptom, or complication, or had a prior diagnosis of diabetes before the current presentation; absence of such documentation was classified as no documented awareness. Travel time to the nearest health facility was based on the estimated one-way travel time recorded in the patient's chart at intake, as assessed by the attending clinician from the patient's place of residence and available means of transportation.

Results — Residence

Consider reporting the distribution of residence in the descriptive tables, since it appears in the regression model but not in the descriptive results.

Response: Residence is now reported in Table 1 (Urban: 143, 36.7%; Rural: 247, 63.3%) in addition to its use in the multivariable model in Table 6, where the category totals now reconcile exactly with Table 1.

Results — Educational status

The educational categories are not mutually exclusive; “Secondary and above” overlaps with “College and above.”

Response: We revised educational status to three mutually exclusive categories that match what is actually recorded in the source data: no formal education, primary, and secondary and above. The overlapping “college and above” category has been removed throughout the manuscript, and a footnote to Table 1 now states explicitly that the categories are mutually exclusive.

Discussion

Greater emphasis should be placed on studies from sub-Saharan Africa and on explaining why hypertension, smoking, and educational status were not independently associated with delayed diagnosis.

Response: We expanded the Discussion paragraph addressing family history, educational status, symptoms, hypertension, and smoking to explicitly compare our null findings with studies from other sub-Saharan African settings, and to offer plausible explanations (differences in population characteristics, sample size and statistical power, residual confounding) while cautioning against over-interpreting these null results as evidence that the factors are unimportant.

Conclusion

Conclusions should avoid strong policy recommendations that imply causal inference.

Response: We revised the Conclusion so that recommendations are explicitly framed as directions supported by the associations observed in this cross-sectional sample, and we added an explicit call for prospective, multicenter research to confirm temporal relationships — including for alcohol use — before stronger causal claims are drawn.

EDITORIAL COMMENTS

Terminology and abbreviations

Ensure consistency in terminology throughout the manuscript and standardize abbreviations.

Response: Terminology (e.g., “facility-based cross-sectional study,” “delayed diagnosis”) and abbreviations (T2DM, FPG, AOR, COR, CI, WUNEMMCSH) have been standardized throughout.

Proofreading

Carefully proofread the manuscript for grammar and punctuation.

Response: The manuscript was proofread in full. This included correcting a garbled, duplicated-clause sentence in the operational-definitions paragraph and a formatting error (duplicated comma) in the author byline.

Numerical consistency across tables, figures, and text

Verify that all tables, figures, and text report identical numerical values.

Response: Every numerical value in the abstract, text, and tables was cross-checked against the underlying dataset. This process uncovered two record-level data problems in the analytic file itself (an implausible age value and a corrupted smoking-history variable), which we traced back to the original paper source forms and prior SPSS output and corrected where independent verification was possible. Where verification was not possible — the smoking term within the Table 6 multivariable model only — we chose transparent disclosure via a table footnote and a note in the Statistical Analysis section rather than presenting an unverified number as confirmed; smoking history is not statistically significant in either version of the model, so this does not affect the study's substantive conclusions.

Table, figure, and reference formatting

Confirm that tables and figures conform to the journal's formatting requirements, and that all references conform to the journal's formatting requirements.

Response: Tables have been reformatted for consistent styling (headers, borders, footnotes) throughout the manuscript. We will gladly conform references and tables to any journal-specific style guide the Editor can provide, as the version submitted follows a generic Vancouver-style format pending confirmation of the target journal's specific requirements.

We thank the Editor and Reviewer again for their thorough and constructive feedback, which has substantially improved the accuracy, transparency, and methodological rigor of this manuscript.

Sincerely,

Million Mohammed Asfaw, M.D. (Corresponding author)

Alemu Earsido Addila, Ph.D.

Attachment Submitted filename: Response to Reviewers.docx

# Peer review: Decision Letter 1

**DOI:** 10.1371/journal.pone.0353573.r003

**Authors:** Mirna Alejandra Gonzalez-Gonzalez

26 Aug 2026

Dear Dr. Million Mohammed Asfaw,

Thank you for submitting your manuscript to PLOS One. After careful consideration, we feel that it has merit but does not fully meet PLOS One’s publication criteria as it currently stands. Therefore, we invite you to submit a revised version of the manuscript that addresses the points raised during the review process.

**ACADEMIC EDITOR:**

Please submit your revised manuscript by  Sep 25 2026 11:59PM. If you will need more time than this to complete your revisions, please reply to this message or contact the journal office at plosone@plos.org. When you're ready to submit your revision, log on to https://www.editorialmanager.com/pone/ and select the 'Submissions Needing Revision' folder to locate your manuscript file.

- A letter that responds to each point raised by the academic editor and reviewer(s). You should upload this letter as a separate file labeled 'Response to Reviewers'.
- A marked-up copy of your manuscript that highlights changes made to the original version. You should upload this as a separate file labeled 'Revised Manuscript with Track Changes'.
- An unmarked version of your revised paper without tracked changes. You should upload this as a separate file labeled 'Manuscript'.

If you would like to make changes to your financial disclosure, please include your updated statement in your cover letter. Guidelines for resubmitting your figure files are available below the reviewer comments at the end of this letter.

If applicable, we recommend that you deposit your laboratory protocols in protocols.io to enhance the reproducibility of your results. Protocols.io assigns your protocol its own identifier (DOI) so that it can be cited independently in the future. For instructions see: https://journals.plos.org/plosone/s/submission-guidelines#loc-laboratory-protocols. Additionally, PLOS One offers an option for publishing peer-reviewed Lab Protocol articles, which describe protocols hosted on protocols.io. Read more information on sharing protocols at https://plos.org/protocols?utm_medium=editorial-email&utm_source=authorletters&utm_campaign=protocols.

As the corresponding author, your ORCID iD is verified in the submission system and will appear in the published article. PLOS supports the use of ORCID, and we encourage all coauthors to register for an ORCID iD and use it as well. Please encourage your coauthors to verify their ORCID iD within the submission system before final acceptance, as unverified ORCID iDs will not appear in the published article. *Only*  the individual author can complete the verification step; PLOS staff *cannot*  verify ORCID iDs on behalf of authors.

We look forward to receiving your revised manuscript.

Kind regards,

Mirna Alejandra Gonzalez-Gonzalez, PhD

Academic Editor

PLOS One

Journal Requirements:

If the reviewer comments include a recommendation to cite specific previously published works, please review and evaluate these publications to determine whether they are relevant and should be cited. There is no requirement to cite these works unless the editor has indicated otherwise.

Please review your reference list to ensure that it is complete and correct. If you have cited papers that have been retracted, please include the rationale for doing so in the manuscript text, or remove these references and replace them with relevant current references. Any changes to the reference list should be mentioned in the rebuttal letter that accompanies your revised manuscript. If you need to cite a retracted article, indicate the article’s retracted status in the References list and also include a citation and full reference for the retraction notice.

Additional Editor Comments:

Please clarify precisely how the smoking-history variable was handled in the final multivariable model given that its individual-level values could not be independently verified. Please provide the number of records affected and explain whether exclusion, recoding, or other data-handling procedures were considered.

[Note: HTML markup is below. Please do not edit.]

Reviewers' comments:

Reviewer's Responses to Questions

**Comments to the Author**

Reviewer #1: (No Response)

Reviewer #3: (No Response)

**********

2. Is the manuscript technically sound, and do the data support the conclusions?

Reviewer #1: (No Response)

Reviewer #3: Yes

**********

3. Has the statistical analysis been performed appropriately and rigorously?

Reviewer #1: Yes

Reviewer #3: Yes

**********

4. Have the authors made all data underlying the findings in their manuscript fully available?

The PLOS Data policy

Reviewer #1: Yes

Reviewer #3: Yes

**********

5. Is the manuscript presented in an intelligible fashion and written in standard English?

Reviewer #1: Yes

Reviewer #3: Yes

**********

Reviewer #1: The manuscript states that variables were screened using a p < 0.25 threshold, yet no bivariable results (crude ORs, CIs, p values) are presented. Providing these results would allow readers to verify which variables met the criterion and ensure the selection process was systematic rather than arbitrary.

The text refers to retaining a nine variable model to preserve a “pre specified” structure, but no protocol, registry entry, or IRB approved document is cited. Clarifying where and when this model was originally specified would strengthen methodological transparency.

The manuscript acknowledges that the three month definition of delayed diagnosis is somewhat arbitrary and that alternative thresholds could alter prevalence and associations. Since no sensitivity analyses were conducted, the findings rely on a single cutoff. Providing justification or sensitivity checks would improve confidence in the stability of results.

Please fix these really important topics. Thank you for all your efforts made to clarify previous details.

Reviewer #3: So far, it's good, you addressed the review comments, just a few modifications

1. Read through the whole document especially where there are hyphens, remove these hyphens and complete the sentences where possible, either with a comma, words and phrase reconstruction,

2. The numbers attached to the authors name should be in superscripts, kindly confirm this via author guidelines.

3. Add corresponding author's email to the title page

4. Standardize the line spacing throughout the text

**********

what does this mean?). If published, this will include your full peer review and any attached files.

If you choose “no”, your identity will remain anonymous but your review may still be made public.

**Do you want your identity to be public for this peer review?**  For information about this choice, including consent withdrawal, please see our Privacy Policy

Reviewer #1: No

Reviewer #3: No

**********

[NOTE: If reviewer comments were submitted as an attachment file, they will be attached to this email and accessible via the submission site. Please log into your account, locate the manuscript record, and check for the action link "View Attachments". If this link does not appear, there are no attachment files.]

To ensure your figures meet our technical requirements, please review our figure guidelines: https://journals.plos.org/plosone/s/figures

You may also use PLOS’s free figure tool, NAAS, to help you prepare publication quality figures: https://journals.plos.org/plosone/s/figures#loc-tools-for-figure-preparation.

NAAS will assess whether your figures meet our technical requirements by comparing each figure against our figure specifications.

# Author comment: Author response to Decision Letter 2

**DOI:** 10.1371/journal.pone.0353573.r004

26 Aug 2026

Regarding the smoking-history variable, As detailed in our attached point-by-point response letter, individual-level smoking values could not be independently verified for any of the 390 analyzed records. We considered recoding (not feasible, as a validated record-level re-entry was not available) and exclusion from the multivariable model (not adopted, since smoking was non-significant in both versions, AOR=0.82, and removing it would not change the substantive conclusions). We adopted retention with full disclosure: Table 6 retains the original file-coded values, Table 5 reports the source-verified aggregate, and this discrepancy is now explicitly flagged in the Statistical Analysis, Table 6 footnote, and Limitations sections. Full details are provided in the attached "Response to Reviewers" letter and reflected in the revised manuscript.

Attachment Submitted filename: Response_to_Reviewers_auresp_2.docx

# Peer review: Decision Letter 2

**DOI:** 10.1371/journal.pone.0353573.r005

**Authors:** Mirna Alejandra Gonzalez-Gonzalez

20 Sep 2026

Prevalence and Associated Factors of Delayed Diagnosis of Type 2 Diabetes Mellitus among Adults Attending a Comprehensive Specialized Hospital in Central Ethiopia: A Facility-Based Cross-Sectional Study

PONE-D-26-31627R2

Dear Dr. Million Mohammed Asfaw,

We’re pleased to inform you that your manuscript has been judged scientifically suitable for publication and will be formally accepted for publication once it meets all outstanding technical requirements.

Within one week, you’ll receive an e-mail detailing the required amendments. When these have been addressed, you’ll receive a formal acceptance letter and your manuscript will be scheduled for publication.

An invoice will be generated when your article is formally accepted. Please note, if your institution has a publishing partnership with PLOS and your article meets the relevant criteria, all or part of your publication costs will be covered. Please make sure your user information is up-to-date by logging into Editorial Manager at Editorial Manager® and clicking the ‘Update My Information' link at the top of the page. For questions related to billing, please contact billing support.

If your institution or institutions have a press office, please notify them about your upcoming paper to help maximize its impact. If they’ll be preparing press materials, please inform our press team as soon as possible -- no later than 48 hours after receiving the formal acceptance. Your manuscript will remain under strict press embargo until 2 pm Eastern Time on the date of publication. For more information, please contact onepress@plos.org.

Kind regards,

Mirna Alejandra Gonzalez-Gonzalez, PhD

Academic Editor

PLOS One

Additional Editor Comments (optional):

Reviewers' comments:

Reviewer's Responses to Questions

**Comments to the Author**

Reviewer #3: All comments have been addressed

**********

2. Is the manuscript technically sound, and do the data support the conclusions?

Reviewer #3: Yes

**********

3. Has the statistical analysis been performed appropriately and rigorously?

Reviewer #3: Yes

**********

4. Have the authors made all data underlying the findings in their manuscript fully available?

The PLOS Data policy

Reviewer #3: Yes

**********

5. Is the manuscript presented in an intelligible fashion and written in standard English?

Reviewer #3: Yes

**********

Reviewer #3: Kudos to the authors for this thorough job, done well and abiding by the ethics of the journal. They should be reminded of fixing the funding statement and conflict of interest subsection.

**********

what does this mean?). If published, this will include your full peer review and any attached files.

If you choose “no”, your identity will remain anonymous but your review may still be made public.

**Do you want your identity to be public for this peer review?**  For information about this choice, including consent withdrawal, please see our Privacy Policy

Reviewer #3: No

**********

# Editor report: Acceptance letter

**DOI:** 10.1371/journal.pone.0353573.r006

**Authors:** Mirna Alejandra Gonzalez-Gonzalez

PONE-D-26-31627R2

PLOS One

Dear Dr. Asfaw,

I'm pleased to inform you that your manuscript has been deemed suitable for publication in PLOS One. Congratulations! Your manuscript is now being handed over to our production team.

At this stage, our production department will prepare your paper for publication. This includes ensuring the following:

* All references, tables, and figures are properly cited

* All relevant supporting information is included in the manuscript submission,

* There are no issues that prevent the paper from being properly typeset

You will receive further instructions from the production team, including instructions on how to review your proof when it is ready. Please keep in mind that we are working through a large volume of accepted articles, so please give us a few days to review your paper and let you know the next and final steps.

Lastly, if your institution or institutions have a press office, please let them know about your upcoming paper now to help maximize its impact. If they'll be preparing press materials, please inform our press team within the next 48 hours. Your manuscript will remain under strict press embargo until 2 pm Eastern Time on the date of publication. For more information, please contact onepress@plos.org.

You will receive an invoice from PLOS for your publication fee after your manuscript has reached the completed accept phase. If you receive an email requesting payment before acceptance or for any other service, this may be a phishing scheme. Learn how to identify phishing emails and protect your accounts at https://explore.plos.org/phishing.

If we can help with anything else, please email us at customercare@plos.org.

Thank you for submitting your work to PLOS One and supporting open access.

Kind regards,

PLOS One Editorial Office Staff

on behalf of

Dr. Mirna Alejandra Gonzalez-Gonzalez

Academic Editor

PLOS One
